# Supplementary material for: Convergent Adaptation in Mitochondria of Phylogenetically Distant Birds: Does it Exist?
Source: Genome Biol Evol. 2021 May 25;13(7):evab113. doi: 10.1093/gbe/evab113 (PMC8271140; doi:10.1093/gbe/evab113)
Supplement: evab113_Supplementary_Data [file evab113_supplementary_data.pdf]

SUPPLEMENTARY MATERIALS

1. TreeWAS results

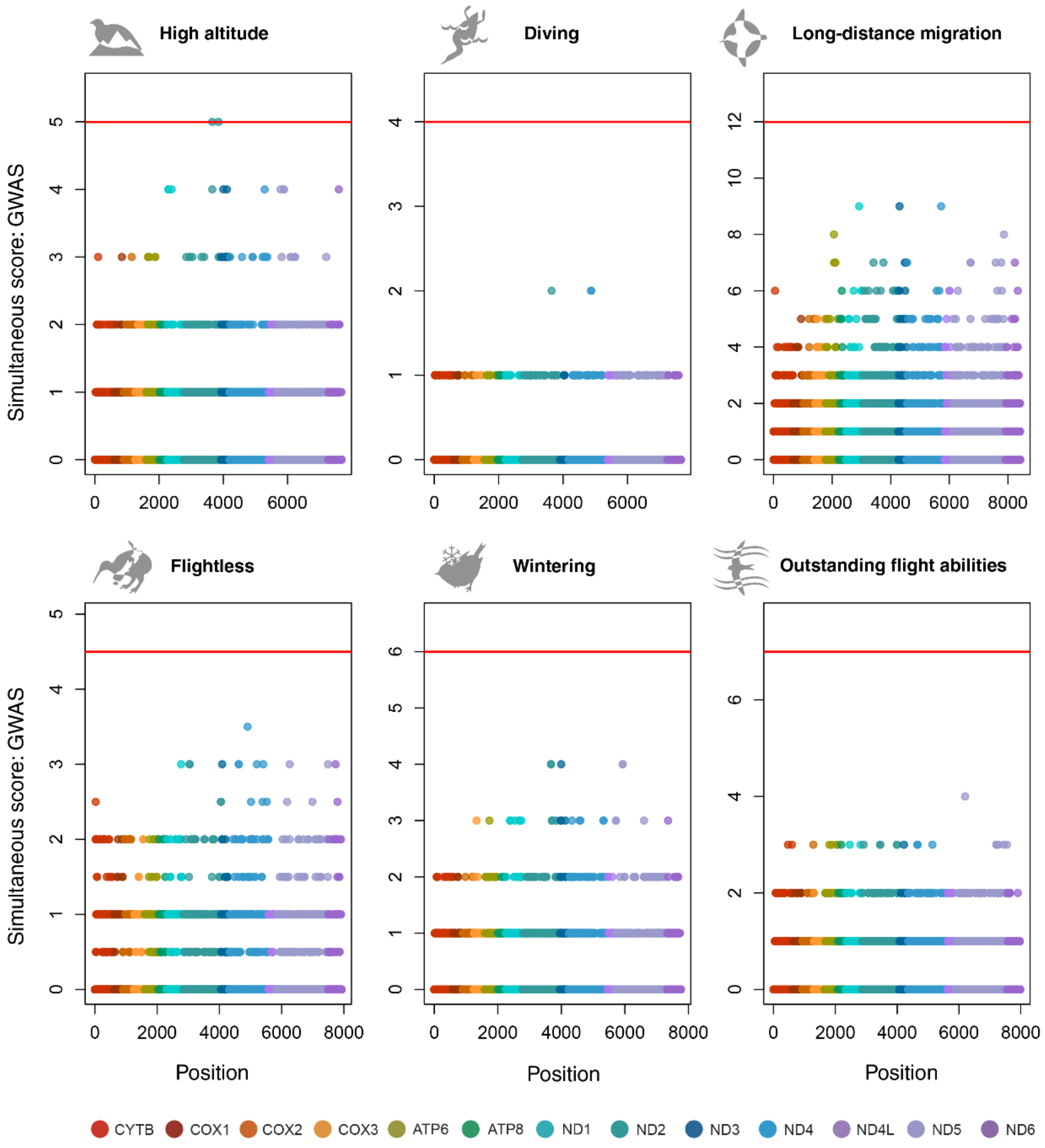

Fig. S1: Simultaneous score. GWAS. Horizontal axis, position in the mitochondrial genes; vertical axis, number of simultaneous changes of phenotype and genotype. Red line

corresponds to significance threshold 0.05 with Bonferroni correction accounting for the number of considered sites in particular test and phenotype (Correction 1).

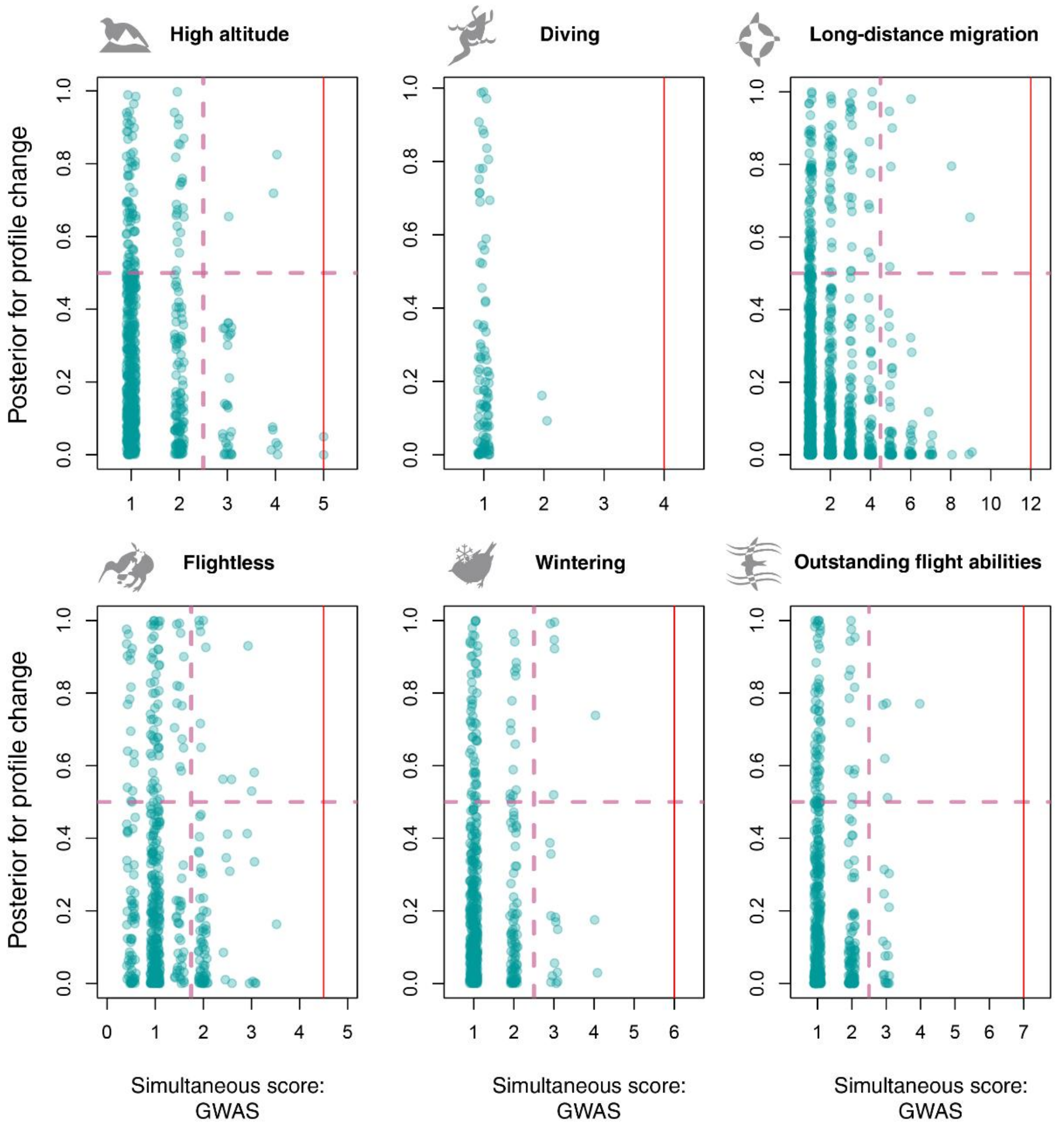

Fig. S2: Profile change vs simultaneous score (GWAS). Red line shows significance threshold for simultaneous test. Dashed lines show division of plot for Fisher test.

a

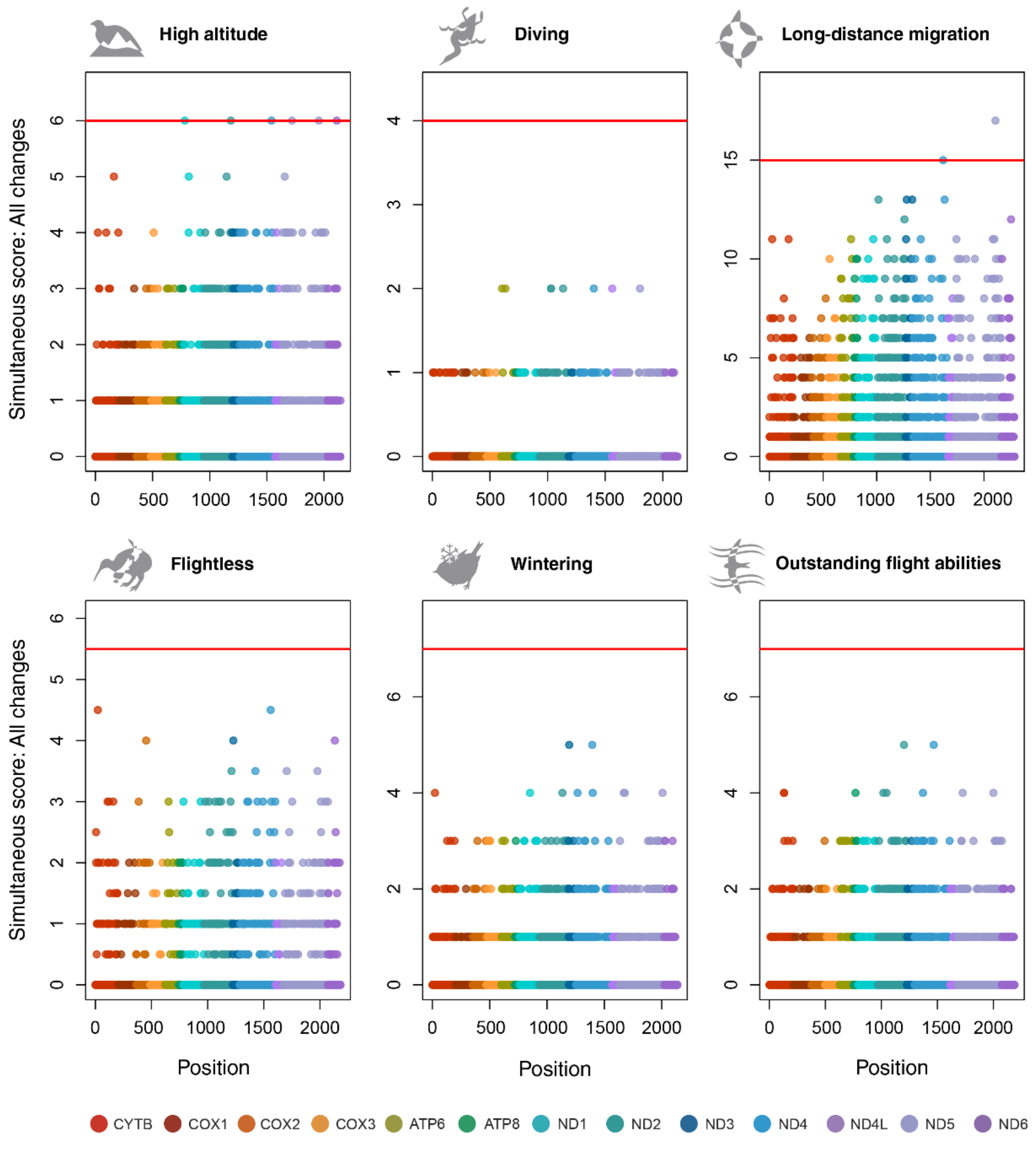

Fig. S3: Simultaneous score. All changes. Horizontal axis, position in the mitochondrial genes; vertical axis, number of simultaneous changes of phenotype and genotype. Red line corresponds to significance threshold 0.05 with Bonferroni correction accounting for the number of considered sites in particular test and phenotype (Correction 1).

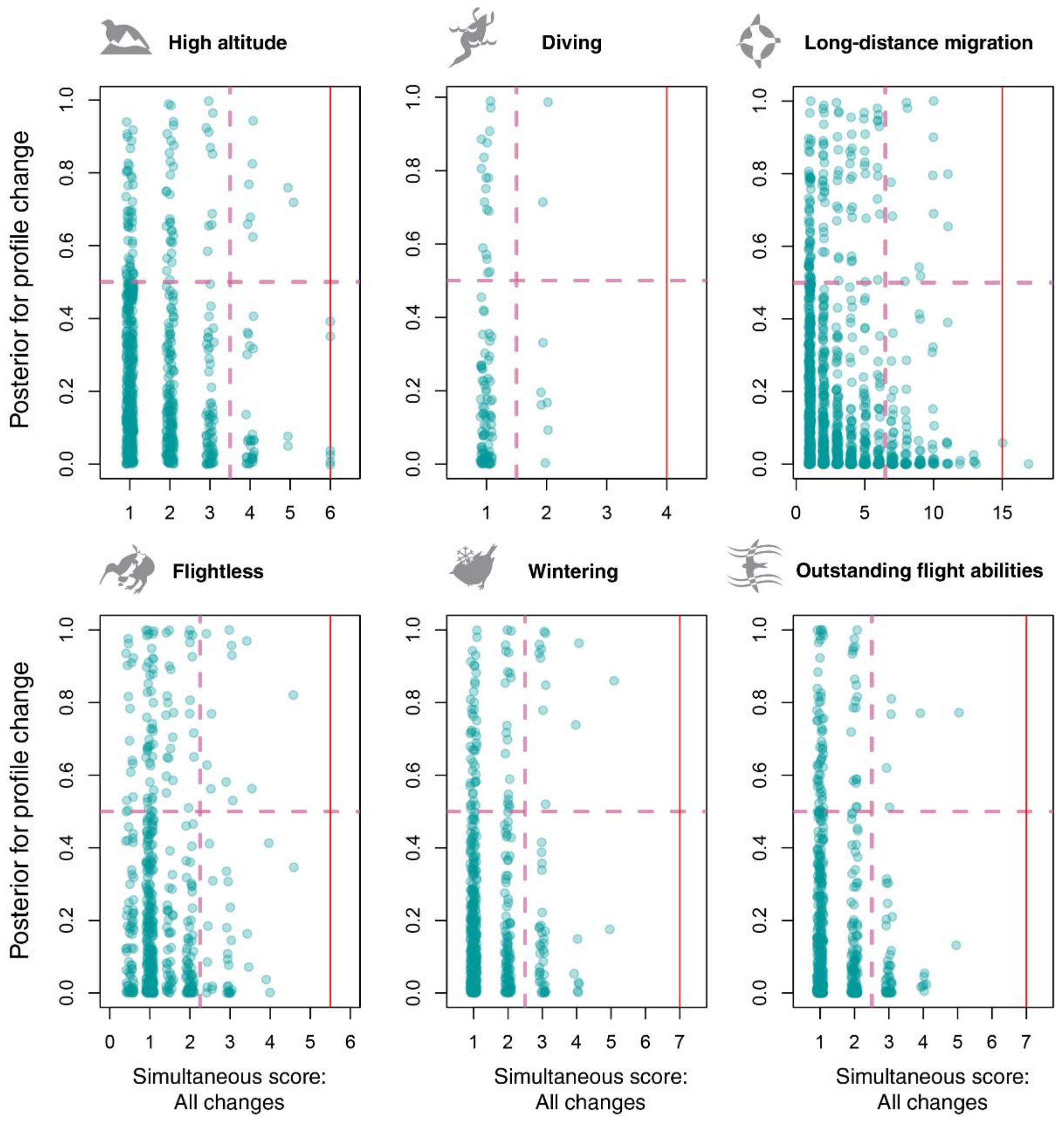

Fig. S4: Profile change vs simultaneous score (All changes). Red line shows significance threshold for simultaneous test. Dashed lines show division of plot for Fisher test.

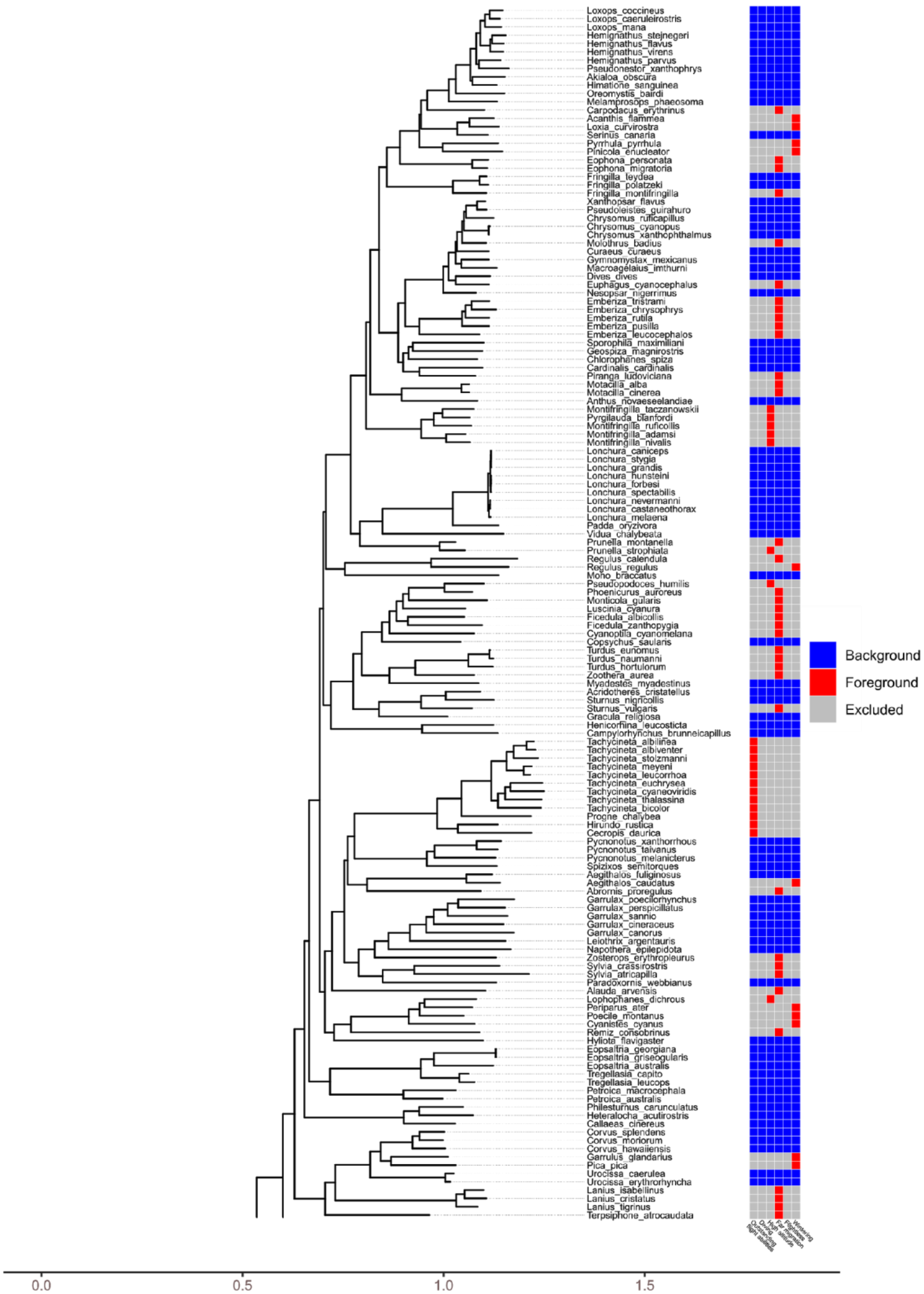
Loxops_coccineus
Loxops_caeruleirostris
Loxops_mana
Hemignathus_stejnegeri
Hemignathus_flavus
Hemignathus_virens
Hemignathus_parvus
Pseudonestor_xanthophrys
Akialoa_obscura
Himatione_sanguinea
Oreomystis_bairdi
Melamprosops_phaeosoma
Carpodacus_erythrinus
Acanthis_flammea
Loxia_curvirostra
Serinus_canaria
Pyrrhula_pyrrhula
Pinicola_enucleator
Eophona_personata
Eophona_migratoria
Fringilla_teydea
Fringilla_polatzeki
Fringilla_montifringilla
Xanthopsar_flavus
Pseudoleistes_guirahuro
Chrysomus_ruficapillus
Chrysomus_cyanopus
Chrysomus_xanthophthalmus
Molothrus_badius
Curaeus_curaeus
Gymnomystax_mexicanus
Macroagelaius_imthurni
Dives_dives
Euphagus_cyanocephalus
Nesopsar_nigerrimus
Emberiza_tristrami
Emberiza_chrysophrys
Emberiza_rutila
Emberiza_pusilla
Emberiza_leucocephalos
Sporophila_maximiliani
Geospiza_magnirostris
Chlorophanes_spiza
Cardinalis_cardinalis
Piranga_ludoviciana
Motacilla_alba
Motacilla_cinerea
Anthus_novaeseelandiae
Montifringilla_taczanowskii
Pyrgilauda_blanfordi
Montifringilla_ruficollis
Montifringilla_adamsi
Montifringilla_nivalis
Lonchura_caniceps
Lonchura_stygia
Lonchura_grandis
Lonchura_hunsteini
Lonchura_forbesi
Lonchura_spectabilis
Lonchura_nevermanni
Lonchura_castaneothorax
Lonchura_melaena
Padda_oryzivora
Vidua_chalybeata
Prunella_montanella
Prunella_strophiata
Regulus_calendula
Regulus_regulus
Moho_braccatus
Pseudopodoces_humilis
Phoenicurus_auroreus
Monticola_gularis
Luscinia_cyanura
Ficedula_albicollis
Ficedula_zanthopygia
Cyanoptila_cyanomelana
Copsychus_saularis
Turdus_eunomus
Turdus_naumanni
Turdus_hortulorum
Zoothera_aurea
Myadestes_myadestinus
Acridotheres_cristatellus
Sturnus_nigricollis
Sturnus_vulgaris
Gracula_religiosa
Henicorhina_leucosticta
Campylorhynchus_brunneicapillus
Tachycineta_albilinea
Tachycineta_albiventer
Tachycineta_stolzmanni
Tachycineta_meyeni
Tachycineta_leucorrhoa
Tachycineta_euchrysea
Tachycineta_cyaneoviridis
Tachycineta_thalassina
Tachycineta_bicolor
Progne_chalybea
Hirundo_rustica
Cecropis_daurica
Pycnonotus_xanthorrhous
Pycnonotus_taivanus
Pycnonotus_melanicterus
Spizixos_semitorques
Aegithalos_fuliginosus
Aegithalos_caudatus
Abrornis_proregulus
Garrulax_poecilorhynchus
Garrulax_perspicillatus
Garrulax_sannio
Garrulax_cineraceus
Garrulax_canorus
Leiothrix_argentauris
Napothera_epilepidota
Zosterops_erythropleurus
Sylvia_crassirostris
Sylvia_atricapilla
Paradoxornis_webbianus
Alauda_arvensis
Lophophanes_dichrous
Periparus_ater
Poecile_montanus
Cyanistes_cyanus
Remiz_consobrinus
Hyliota_flavigaster
Eopsaltria_georgiana
Eopsaltria_griseogularis
Eopsaltria_australis
Tregellasia_capito
Tregellasia_leucops
Petroica_macrocephala
Petroica_australis
Philesturnus_carunculatus
Heteralocha_acutirostris
Callaeas_cinereus
Corvus_splendens
Corvus_moriorum
Corvus_hawaiiensis
Garrulus_glandarius
Pica_pica
Urocissa_caerulea
Urocissa_erythrorhyncha
Lanius_isabellinus
Lanius_cristatus
Lanius_tigrinus
Terpsiphone_atrocaudata
Outstanding flight abilities
Diving
High altitude
Far migration
Flightless
Wintering
Background
Foreground
Excluded
0.0
0.5
1.0
1.5

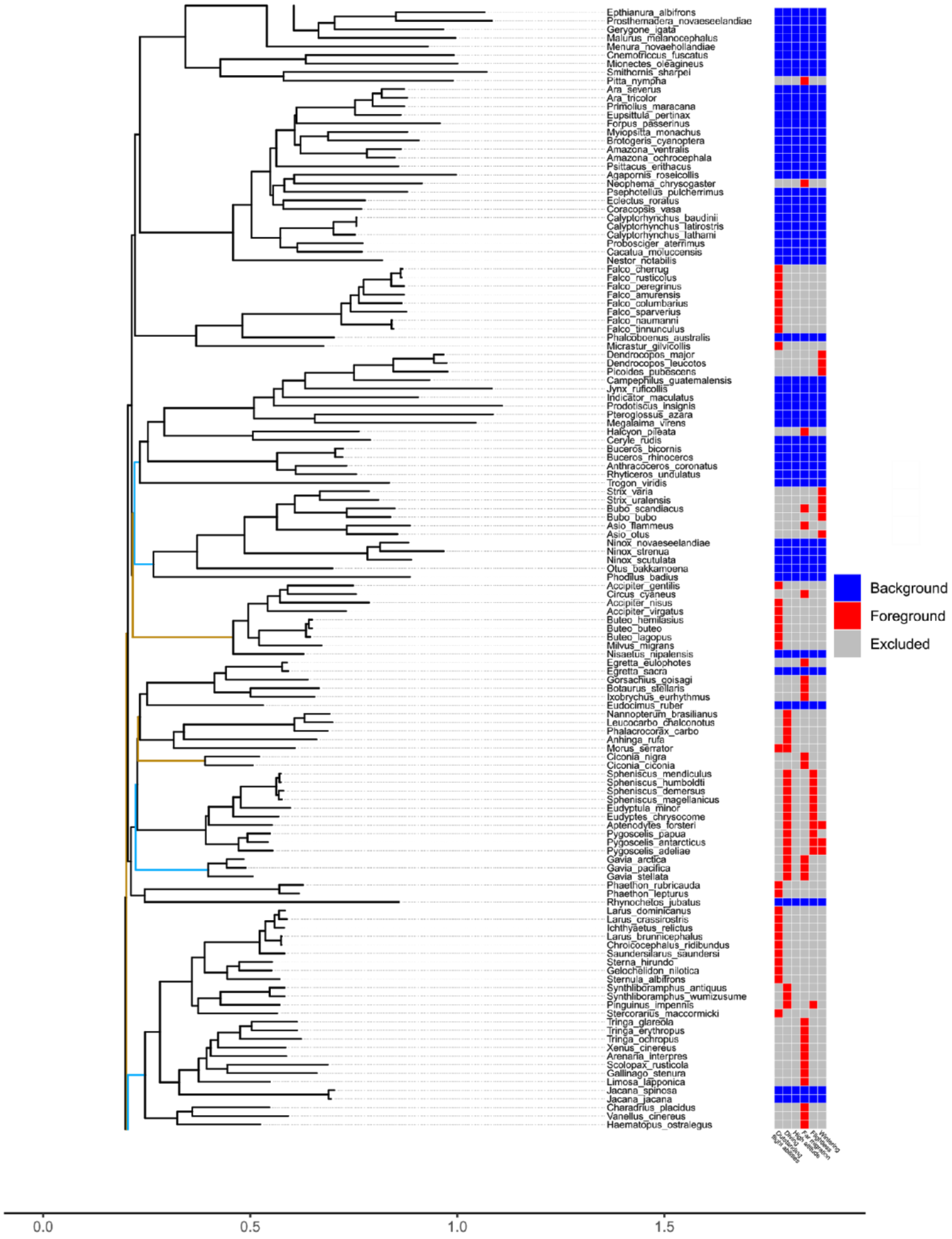

Epthianura_albifrons
Prosthemadera_novaeseelandiae
Gerygone_igata
Malurus_melanocephalus
Menura_novaehollandiae
Cnemotriccus_fuscatus
Mionectes_oleagineus
Smithornis_sharpei
Pitta_nympha
Ara_severus
Ara_tricolor
Primolius_maracana
Eupsittula_pertinax
Forpus_passerinus
Myiopsitta_monachus
Brotogeris_cyanoptera
Amazona_ventralis
Amazona_ochrocephala
Psittacus_erithacus
Agapornis_roseicollis
Neophema_chrysogaster
Psephotellus_pulcherrimus
Eclectus_roratus
Coracopsis_vasa
Calyptorhynchus_baudinii
Calyptorhynchus_latirostris
Calyptorhynchus_lathami
Probosciger_aterrimus
Cacatua_moluccensis
Nestor_notabilis
Falco_cherrug
Falco_rusticolus
Falco_peregrinus
Falco_amurensis
Falco_columbarius
Falco_sparverius
Falco_naumanni
Falco_tinnunculus
Phalcoboenus_australis
Micrastur_gilvicollis
Dendrocopos_major
Dendrocopos_leucotos
Picoides_pubescens
Campephilus_guatemalensis
Jynx_ruficollis
Indicator_maculatus
Prodotiscus_insignis
Pteroglossus_azara
Megalaima_virens
Halcyon_pileata
Ceryle_rudis
Buceros_bicornis
Buceros_rhinoceros
Anthracoceros_coronatus
Rhyticeros_undulatus
Trogon_viridis
Strix_varia
Strix_uralensis
Bubo_scandiacus
Bubo_bubo
Asio_flammeus
Asio_otus
Ninox_novaeseelandiae
Ninox_strenua
Ninox_scutulata
Otus_bakkamoena
Phodilus_badius
Accipiter_gentilis
Circus_cyaneus
Accipiter_nisus
Accipiter_virgatus
Buteo_hemilasius
Buteo_buteo
Buteo_lagopus
Milvus_migrans
Nisaetus_nipalensis
Egretta_eulophotes
Egretta_sacra
Gorsachius_goisagi
Botaurus_stellaris
Ixobrychus_eurhythmus
Eudocimus_ruber
Nannopterum_brasilianus
Leucocarbo_chalconotus
Phalacrocorax_carbo
Anhinga_rufa
Morus_serrator
Ciconia_nigra
Ciconia_ciconia
Spheniscus_mendiculus
Spheniscus_humboldti
Spheniscus_demersus
Spheniscus_magellanicus
Eudyptula_minor
Eudyptes_chrysocome
Aptenodytes_forsteri
Pygoscelis_papua
Pygoscelis_antarcticus
Pygoscelis_adeliae
Gavia_arctica
Gavia_pacifica
Gavia_stellata
Phaethon_rubricauda
Phaethon_lepturus
Rhynochetos_jubatus
Larus_dominicanus
Larus_crassirostris
Ichthyaetus_relictus
Larus_brunnicephalus
Chroicocephalus_ridibundus
Saundersilarus_saundersi
Sterna_hirundo
Gelochelidon_nilotica
Sternula_albifrons
Synthliboramphus_antiquus
Synthliboramphus_wumizusume
Pinguinus_impennis
Stercorarius_maccormicki
Tringa_glareola
Tringa_erythropus
Tringa_ochropus
Xenus_cinereus
Arenaria_interpres
Scolopax_rusticola
Gallinago_stenura
Limosa_lapponica
Jacana_spinosa
Jacana_jacana
Charadrius_placidus
Vanellus_cinereus
Haematopus_ostralegus
Outstanding flight abilities
Diving
High altitude
Far migration
Flightless
Wintering
Background
Foreground
Excluded
0.0
0.5
1.0
1.5

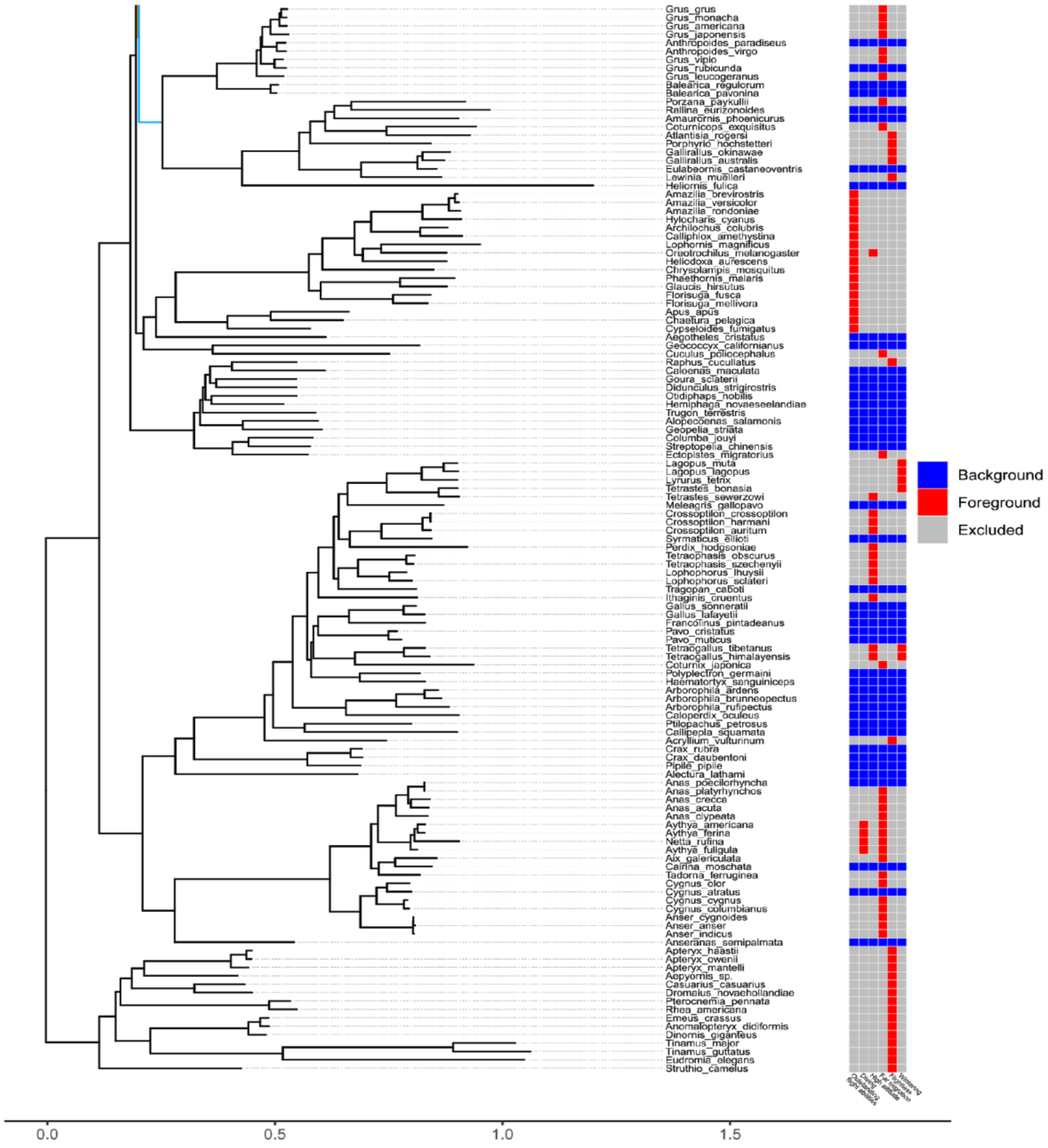

Fig. S5: Phylogentic tree and phenotype groups. Distances are measured in number of substitutions per nucleotide site.

2. PCOC results

TreeWAS method is fairly criticized for inattention to differences of evolutionary rates in different sites. To test alternative approach, we used PCOC (Rey et al 2018), which is designed for amino acid convergent evolution detection.

It takes advantage of complex evolutionary models and Bayesian parameter optimization. It implements site heterogeneity model (Gamma distribution) and should be robust when evaluating convergence at hypervariable sites. The PCOC consists of three tests. First test, called PC (Profile Change), estimates changes of amino acid site-specific propensities between foreground and background phenotype (we also used it earlier in this work). Second method, called OC (One change) relies on synchronous changes of phenotype and genotype at the tree. It accounts for changes between any two amino acids regardless of their association with phenotype. This method is essentially close to “All changes” modification of Simultaneous score method in TreeWAS. Third method, called PCOC, unites PC and OC tests to give overall estimation of convergence probability at the site. PCOC is known to perform better than any of these methods individually. Here we consider only sites with high scores for all three tests: posterior probabilities in PC test should be higher than 0.90, posterior probabilities in OC and PCOC tests should be higher than 0.99. All six phenotypes were tested.

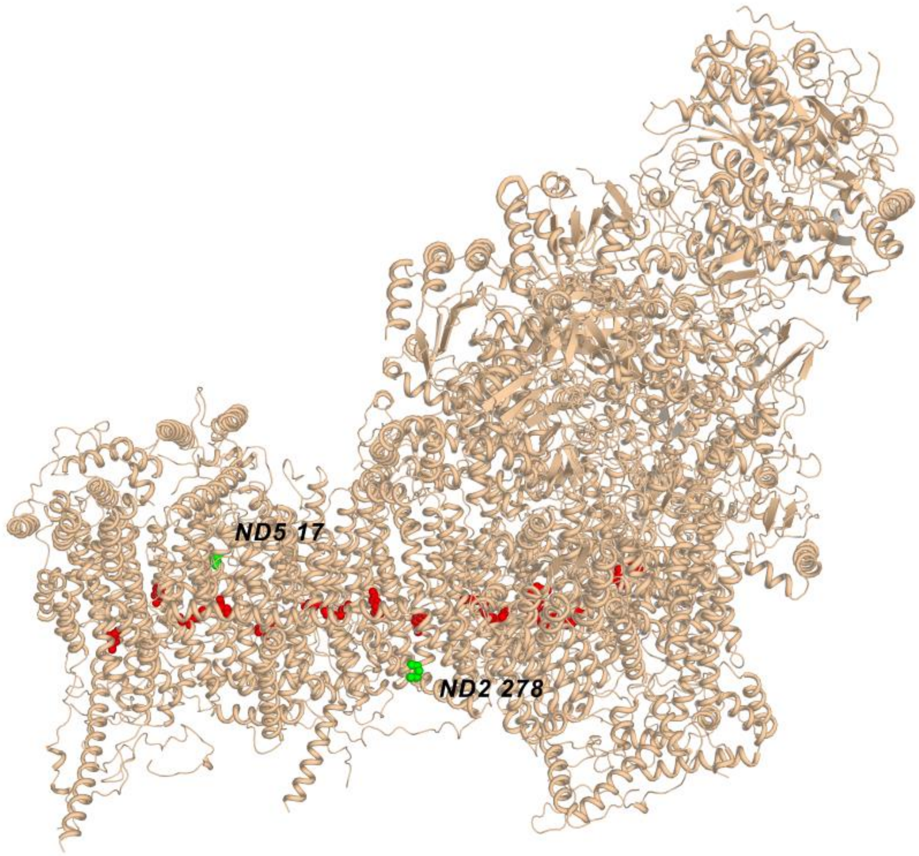

Fig. S6: 3D structure of respiratory complex I. Candidate amino acid residues, detected by PCOC tool are colored green. Polar residues in proton channels, which play a key role in proton transport, are colored red.

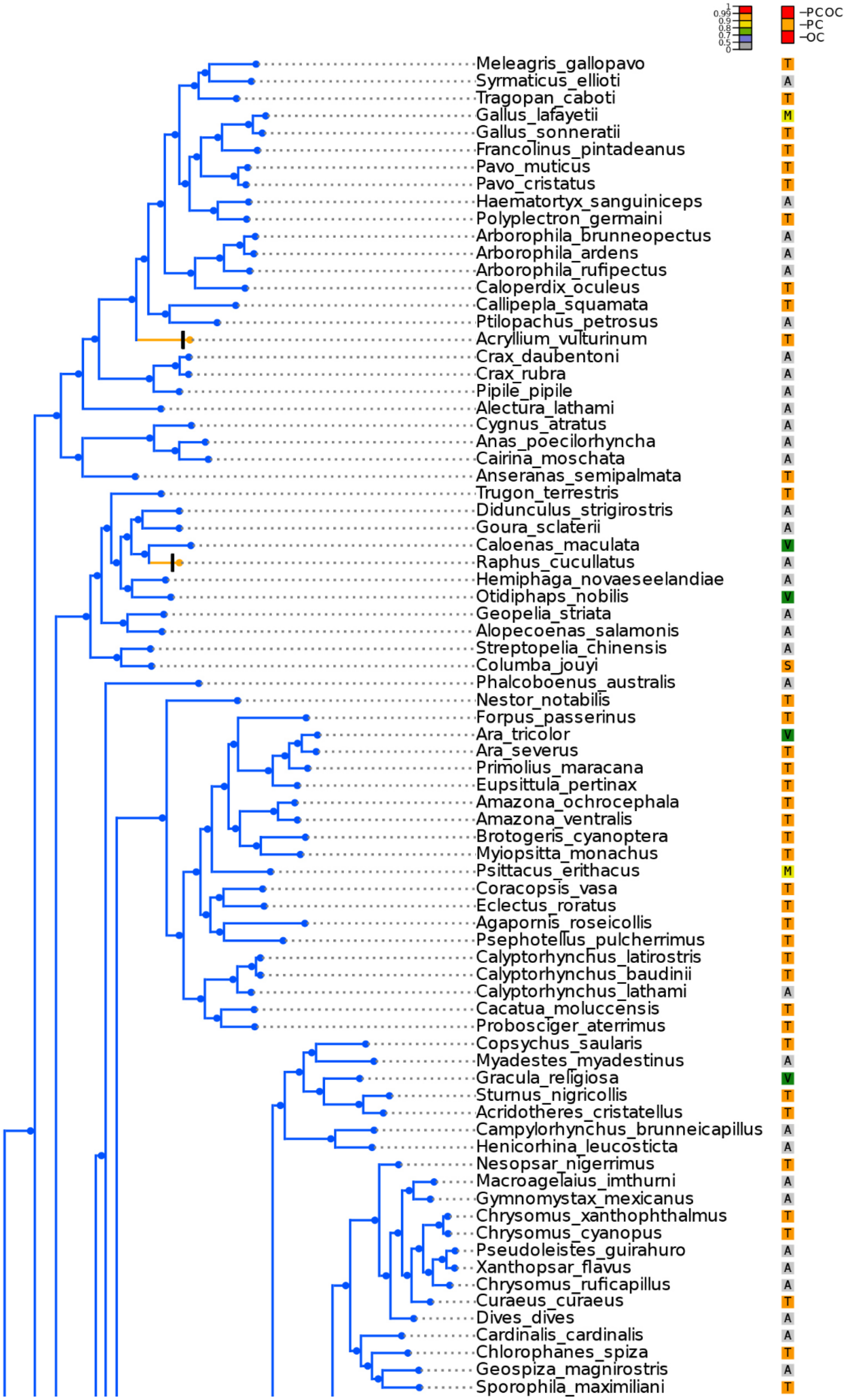

1
0.99
0.9
0.8
0.7
0.5
0
PCOC
PC
OC
Meleagris_gallopavo T
Syrmaticus_ellioti A
Tragopan_caboti T
Gallus_lafayetii M
Gallus_sonneratii T
Francolinus_pintadeanus T
Pavo_muticus T
Pavo_cristatus T
Haematortyx_sanguiniceps A
Polyplectron_germaini T
Arborophila_brunneopectus A
Arborophila_ardens A
Arborophila_rufipectus A
Caloperdix_oculeus T
Callipepla_squamata T
Ptilopachus_petrosus A
Acryllium_vulturinum T
Crax_daubentoni A
Crax_rubra A
Pipile_pipile A
Alectura_lathami A
Cygnus_atratus A
Anas_poecilorhyncha A
Cairina_moschata A
Anseranas_semipalmata T
Trugon_terrestris T
Didunculus_strigirostris A
Goura_sclaterii A
Caloenas_maculata V
Raphus_cucullatus A
Hemiphaga_novaeseelandiae A
Otidiphaps_nobilis V
Geopelia_striata A
Alopecoenas_salamonis A
Streptopelia_chinensis A
Columba_jouyi S
Phalcoboenus_australis A
Nestor_notabilis T
Forpus_passerinus T
Ara_tricolor V
Ara_severus T
Primolius_maracana T
Eupsittula_pertinax T
Amazona_ochrocephala T
Amazona_ventralis T
Brotogeris_cyanoptera T
Myiopsitta_monachus T
Psittacus_erithacus M
Coracopsis_vasa T
Eclectus_roratus T
Agapornis_roseicollis T
Psephotellus_pulcherrimus T
Calyptorhynchus_latirostris T
Calyptorhynchus_baudinii T
Calyptorhynchus_lathami A
Cacatua_moluccensis T
Probosciger_aterrimus T
Copsychus_saularis T
Myadestes_myadestinus A
Gracula_religiosa V
Sturnus_nigricollis T
Acridotheres_cristatellus T
Campylorhynchus_brunneicapillus A
Henicorhina_leucosticta A
Nesopsar_nigerrimus T
Macroagelaius_imthurni A
Gymnomystax_mexicanus A
Chrysomus_xanthophthalmus T
Chrysomus_cyanopus T
Pseudoleistes_guirahuro A
Xanthopsar_flavus A
Chrysomus_ruficapillus A
Curaeus_curaeus T
Dives_dives A
Cardinalis_cardinalis A
Chlorophanes_spiza T
Geospiza_magnirostris A
Sporophila_maximiliani T

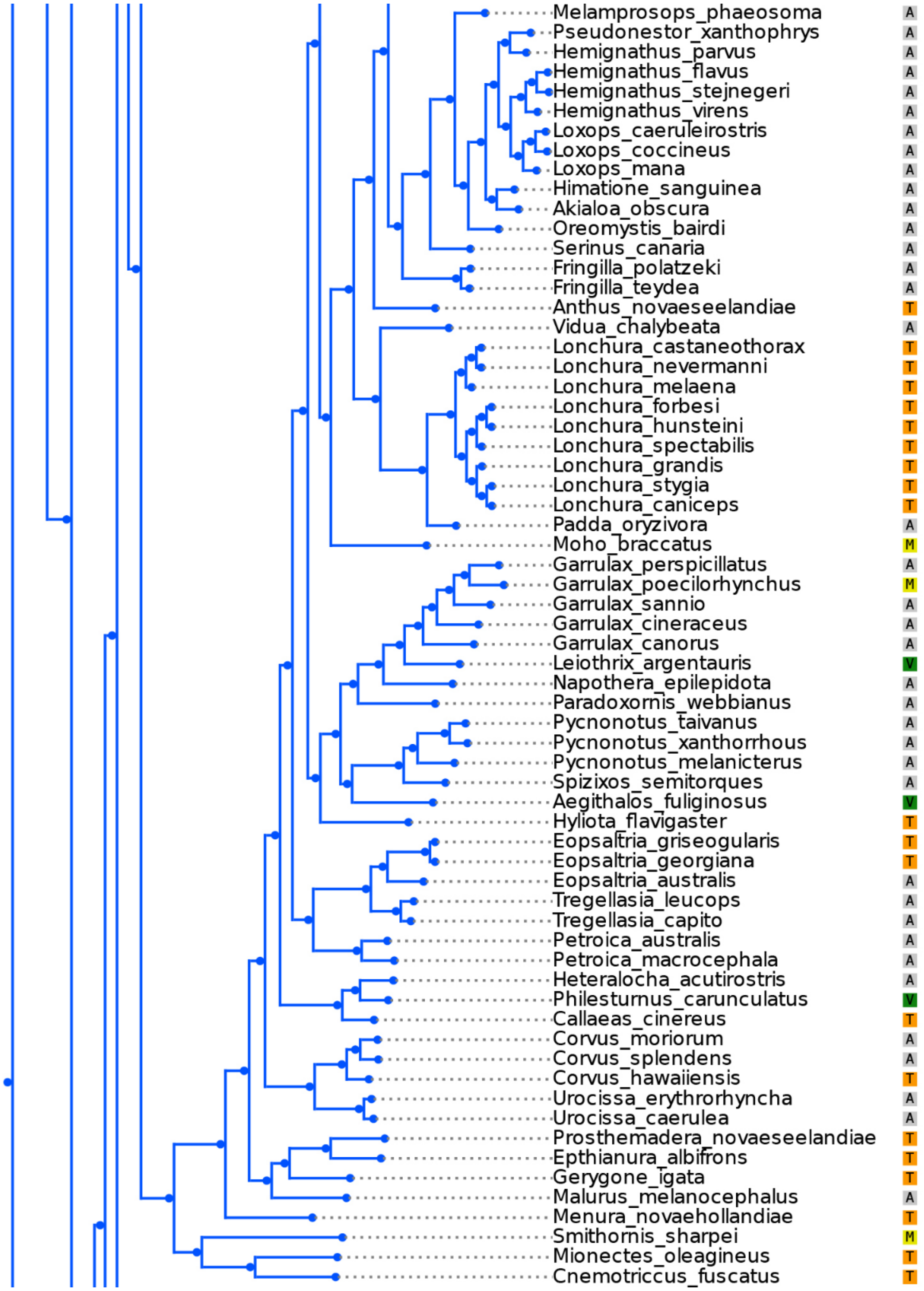

Melamprosops_phaeosoma A
Pseudonestor_xanthophrys A
Hemignathus_parvus A
Hemignathus_flavus A
Hemignathus_stejnegeri A
Hemignathus_virens A
Loxops_caeruleirostris A
Loxops_coccineus A
Loxops_mana A
Himatione_sanguinea A
Akialoa_obscura A
Oreomystis_bairdi A
Serinus_canaria A
Fringilla_polatzeki A
Fringilla_teydea A
Anthus_novaeseelandiae T
Vidua_chalybeata A
Lonchura_castaneothorax T
Lonchura_nevermanni T
Lonchura_melaena T
Lonchura_forbesi T
Lonchura_hunsteini T
Lonchura_spectabilis T
Lonchura_grandis T
Lonchura_stygia T
Lonchura_caniceps T
Padda_oryzivora A
Moho_braccatus M
Garrulax_perspicillatus A
Garrulax_poecilorhynchus M
Garrulax_sannio A
Garrulax_cineraceus A
Garrulax_canorus A
Leiothrix_argentauris V
Napothera_epilepidota A
Paradoxornis_webbianus A
Pycnonotus_taivanus A
Pycnonotus_xanthorrhous A
Pycnonotus_melanicterus A
Spizixos_semitorques A
Aegithalos_fuliginosus V
Hyliota_flavigaster T
Eopsaltria_griseogularis T
Eopsaltria_georgiana T
Eopsaltria_australis A
Tregellasia_leucops A
Tregellasia_capito A
Petroica_australis A
Petroica_macrocephala A
Heteralocha_acutirostris A
Philesturnus_carunculatus V
Callaeas_cinereus T
Corvus_moriorum A
Corvus_splendens A
Corvus_hawaiiensis T
Urocissa_erythrorhyncha A
Urocissa_caerulea A
Prosthemadera_novaeseelandiae T
Epthianura_albifrons T
Gerygone_igata T
Malurus_melanocephalus A
Menura_novaehollandiae T
Smithornis_sharpei M
Mionectes_oleagineus T
Cnemotriccus_fuscatus T

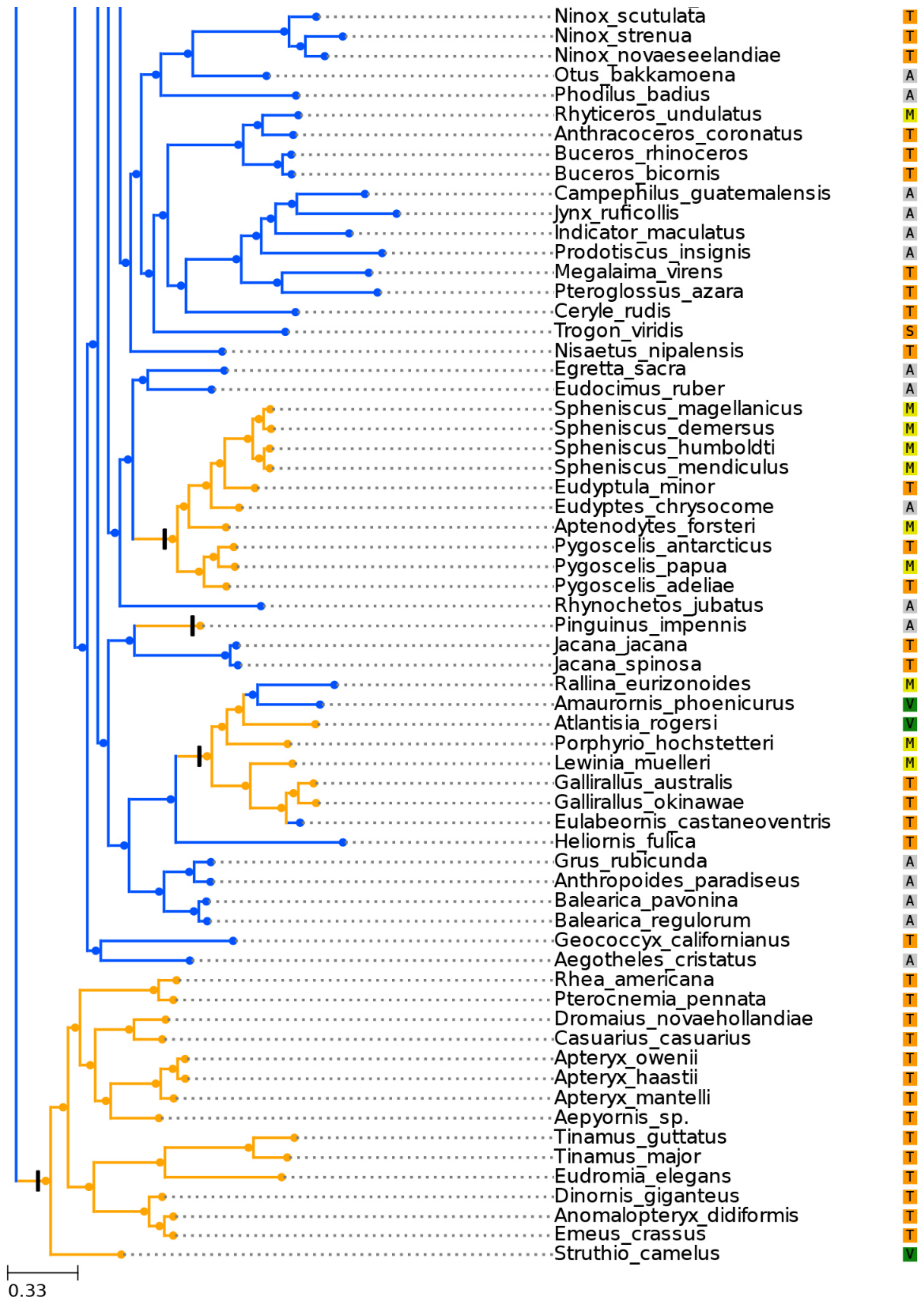

Fig. S7: Position 278 in the *ND2* gene associated with loss of flight.

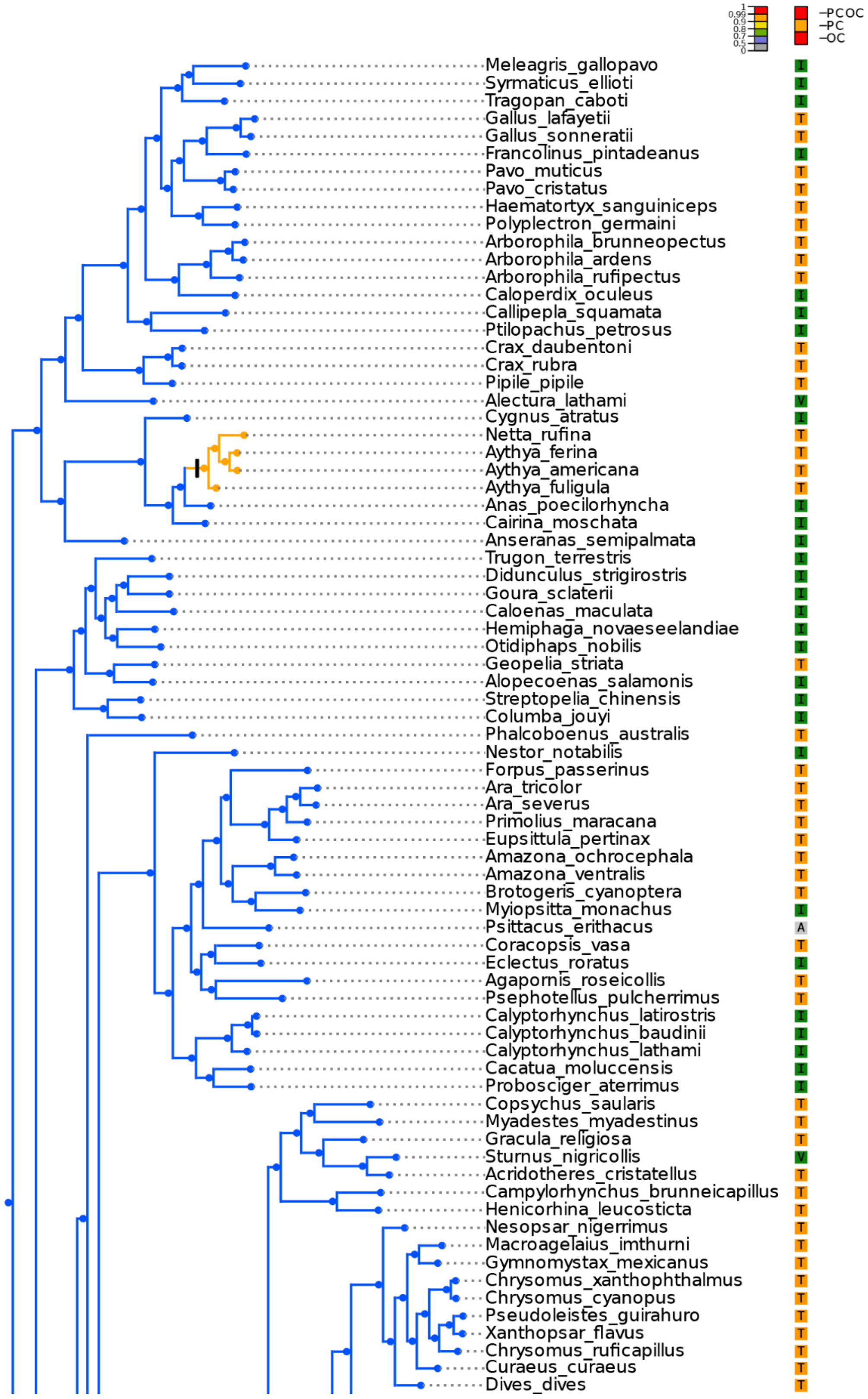
1
0.99
0.9
0.8
0.7
0.5
0
PCOC
PC
OC
Meleagris_gallopavo
Syrmaticus_ellioti
Tragopan_caboti
Gallus_lafayetii
Gallus_sonneratii
Francolinus_pintadeanus
Pavo_muticus
Pavo_cristatus
Haematortyx_sanguiniceps
Polyplectron_germaini
Arborophila_brunneopectus
Arborophila_ardens
Arborophila_rufipectus
Caloperdix_oculeus
Callipepla_squamata
Ptilopachus_petrosus
Crax_daubentoni
Crax_rubra
Pipile_pipile
Alectura_lathami
Cygnus_atratus
Netta_rufina
Aythya_ferina
Aythya_americana
Aythya_fuligula
Anas_poecilorhyncha
Cairina_moschata
Anseranas_semipalmata
Trugon_terrestris
Didunculus_strigirostris
Goura_sclaterii
Caloenas_maculata
Hemiphaga_novaeseelandiae
Otidiphaps_nobilis
Geopelia_striata
Alopecoenas_salamonis
Streptopelia_chinensis
Columba_jouyi
Phalcoboenus_australis
Nestor_notabilis
Forpus_passerinus
Ara_tricolor
Ara_severus
Primolius_maracana
Eupsittula_pertinax
Amazona_ochrocephala
Amazona_ventralis
Brotogeris_cyanoptera
Myiopsitta_monachus
Psittacus_erithacus
Coracopsis_vasa
Eclectus_roratus
Agapornis_roseicollis
Psephotellus_pulcherrimus
Calyptorhynchus_latirostris
Calyptorhynchus_baudinii
Calyptorhynchus_lathami
Cacatua_moluccensis
Probosciger_aterrimus
Copsychus_saularis
Myadestes_myadestinus
Gracula_religiosa
Sturnus_nigricollis
Acridotheres_cristatellus
Campylorhynchus_brunneicapillus
Henicorhina_leucosticta
Nesopsar_nigerrimus
Macroagelaius_imthurni
Gymnomystax_mexicanus
Chrysomus_xanthophthalmus
Chrysomus_cyanopus
Pseudoleistes_guirahuro
Xanthopsar_flavus
Chrysomus_ruficapillus
Curaeus_curaeus
Dives_dives

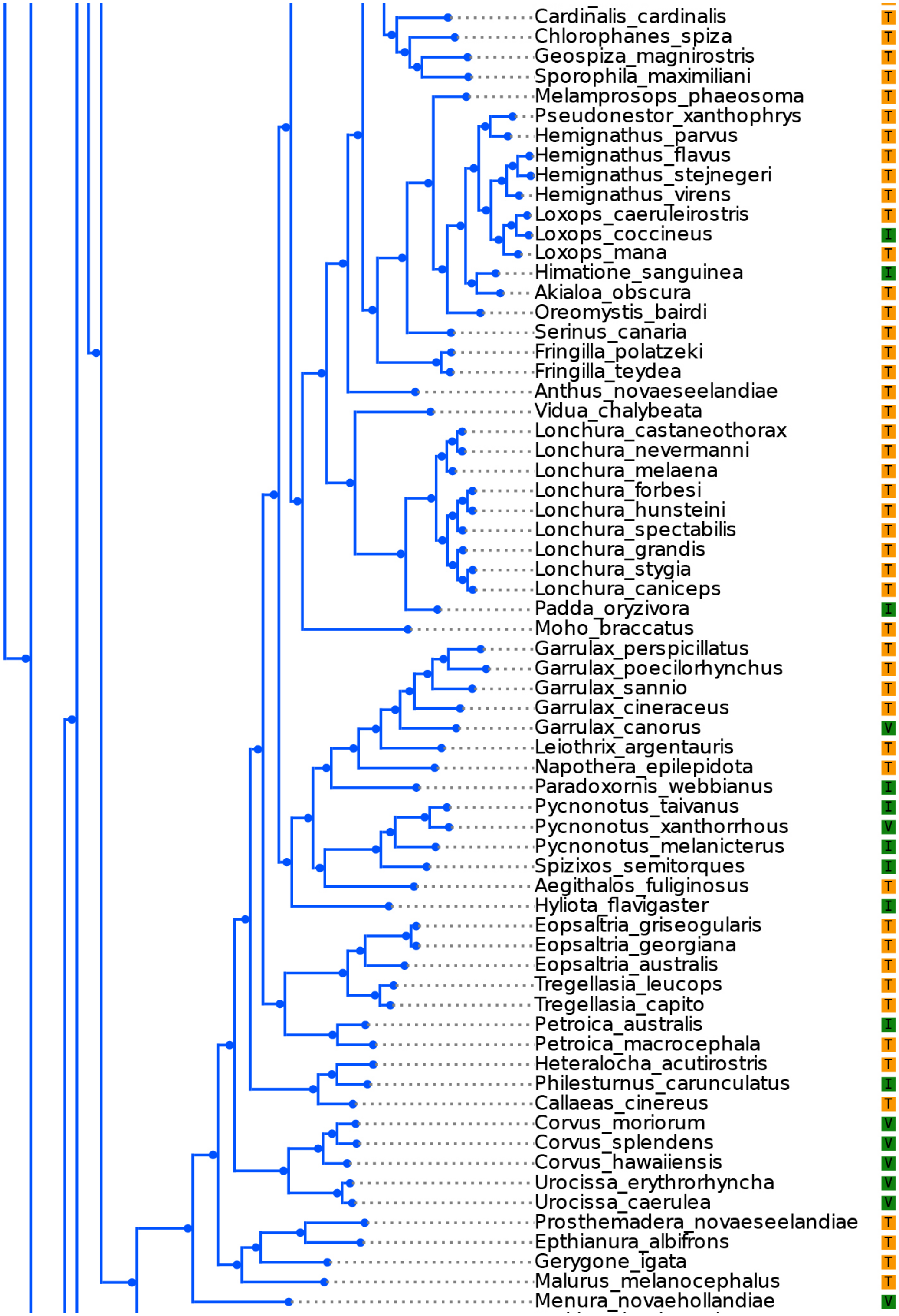
Cardinalis_cardinalis T
Chlorophanes_spiza T
Geospiza_magnirostris T
Sporophila_maximiliani T
Melamprosops_phaeosoma T
Pseudonestor_xanthophrys T
Hemignathus_parvus T
Hemignathus_flavus T
Hemignathus_stejnegeri T
Hemignathus_virens T
Loxops_caeruleirostris T
Loxops_coccineus I
Loxops_mana T
Himatione_sanguinea I
Akialoa_obscura T
Oreomystis_bairdi T
Serinus_canaria T
Fringilla_polatzeki T
Fringilla_teydea T
Anthus_novaeseelandiae T
Vidua_chalybeata T
Lonchura_castaneothorax T
Lonchura_nevermanni T
Lonchura_melaena T
Lonchura_forbesi T
Lonchura_hunsteini T
Lonchura_spectabilis T
Lonchura_grandis T
Lonchura_stygia T
Lonchura_caniceps T
Padda_oryzivora I
Moho_braccatus T
Garrulax_perspicillatus T
Garrulax_poecilorhynchus T
Garrulax_sannio T
Garrulax_cineraceus T
Garrulax_canorus V
Leiothrix_argentauris T
Napothera_epilepidota T
Paradoxornis_webbianus I
Pycnonotus_taivanus I
Pycnonotus_xanthorrhous V
Pycnonotus_melanicterus I
Spizixos_semitorques I
Aegithalos_fuliginosus T
Hyliota_flavigaster I
Eopsaltria_griseogularis T
Eopsaltria_georgiana T
Eopsaltria_australis T
Tregellasia_leucops T
Tregellasia_capito T
Petroica_australis I
Petroica_macrocephala T
Heteralocha_acutirostris T
Philesturnus_carunculatus I
Callaeas_cinereus T
Corvus_moriorum V
Corvus_splendens V
Corvus_hawaiiensis V
Urocissa_erythrorhyncha V
Urocissa_caerulea V
Prosthemadera_novaeseelandiae T
Epthianura_albifrons T
Gerygone_igata T
Malurus_melanocephalus T
Menura_novaehollandiae V

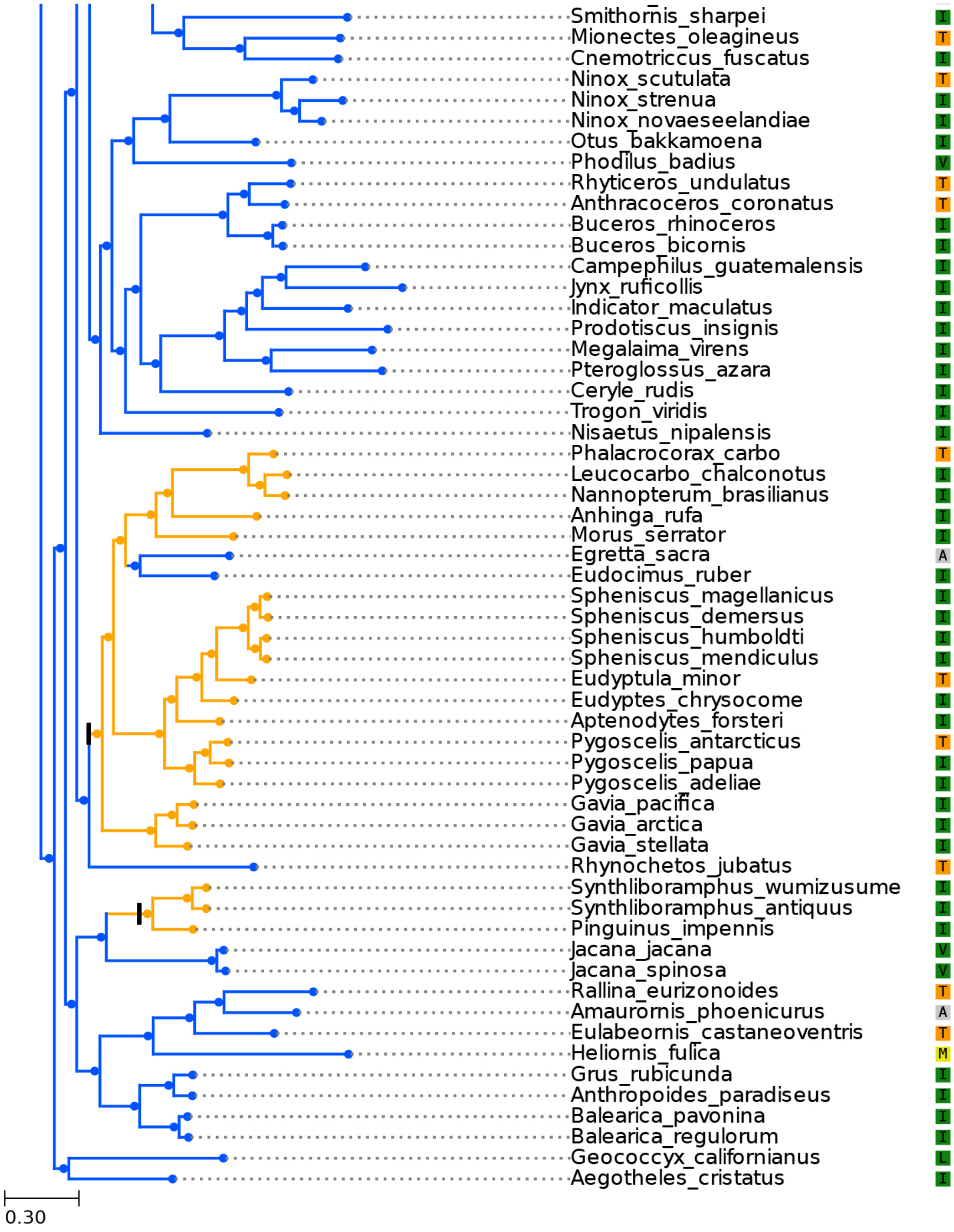

Fig. S8: Position 17 in the *ND5* gene associated with diving.
